# Late 1800s Fringe Electrotherapeutic Devices: Comparative Electrical Capabilities

**DOI:** 10.1192/j.eurpsy.2022.672

**Published:** 2022-09-01

**Authors:** D. Cox, B. Carr

**Affiliations:** 1 New York Institute of Technology, College Of Osteopathic Medicine, Jonesboro, United States of America; 2 University of Florida College of Medicine, Department Of Psychiatry, Gainesville, United States of America

**Keywords:** electrostimulation, device, historyofmedicine

## Abstract

**Introduction:**

Desperation for cure led to 19^th^ century invention-- electrotherapeutic devices; replete with hyperbolic claims of cure-all, perceived ineffectiveness, and potential harm rendered the modality as quackery but were used in early brain stimulation, melancholia treatment, and cortex mapping. Here, antique devices are restored, and their electrophysiological qualities ascertained.

**Objectives:**

Determine the comparative capabilities of these devices in delivering electrostimulation and compare with modern standards to understand possible electrophysiological sequelae.

**Methods:**

Devices known as “medical batteries” were analyzed. Power delivery utilized a “voltaic battery”, simple circuit, and a conductor wrapped around an iron core. When the circuit is energized, the core is magnetized by direct current of the battery which induces an alternating current that electrifies probes used on the body. Due to their marked age, a common 9-volt battery was exchanged for the corrosive dry cell paste batteries. Electrical parameters were then measured.

**Figure:**
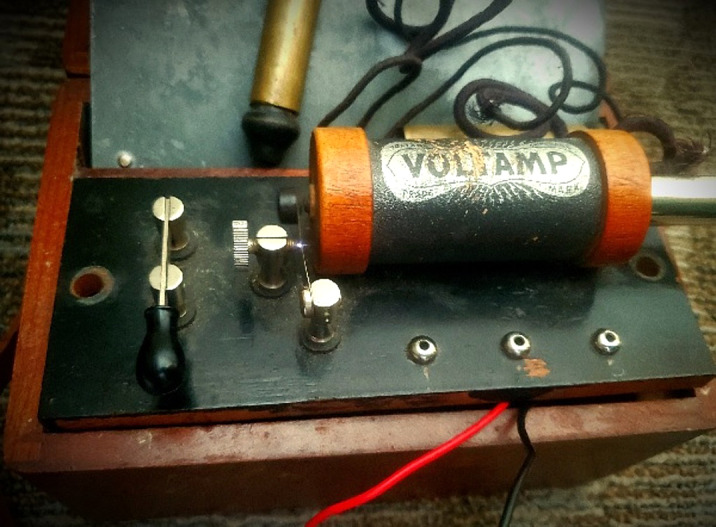

**Figure:**
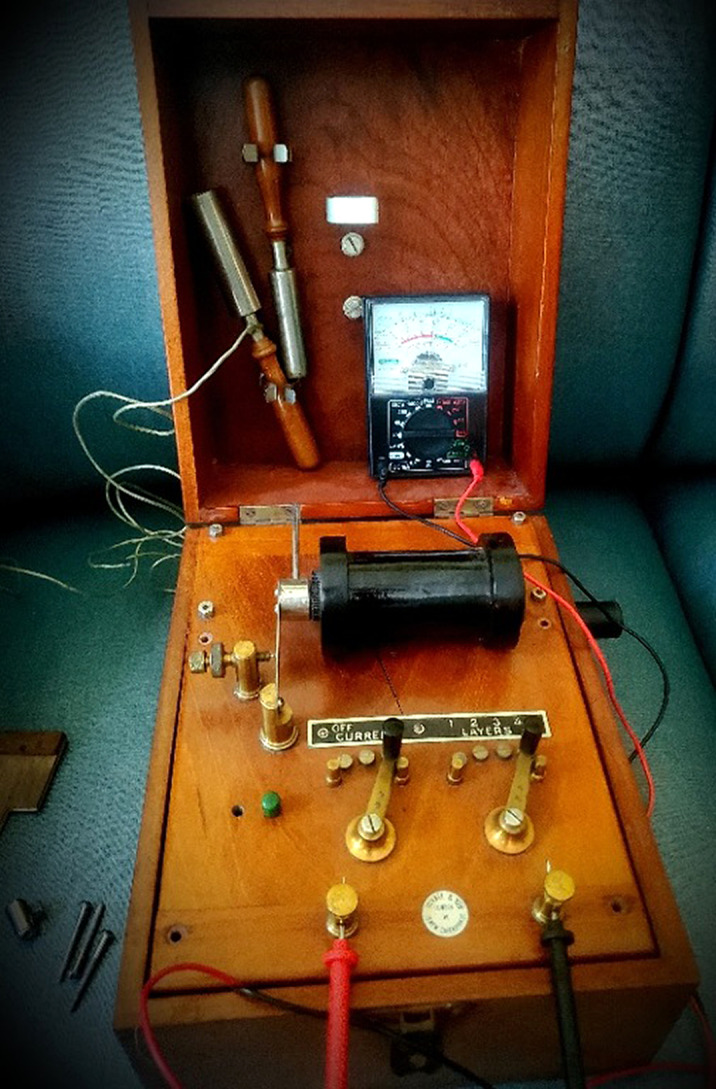

**Results:**

**Table 1:** 

Device	Frequency (Hz)	Resistance (Ohms)	Max Output (Amps)	Min Output (Amps)	Max Output (Volts)	Min Output (Volts)
Voltamp^a^	2k – 12K	60	0.66	0.33	60V	20V
J.H. Bunnell & Co.’s No. 4 D.D.	7k-10k	50	6	0.4	300V	20V
Schall & Son (London)^b^	300-1200	40	10.5	2.75	420V	110V

**Conclusions:**

Devices for electrotherapeutics ranged from anemic vibrations to dangerous tetany inducing shocks. Measuring the capabilities of these devices shows the robust yields possible if the original higher capacity batteries were utilized. The reality is, cure or not, the devices were surprisingly potent. It is interesting that, albeit unrefined, efficacious doses were available before modern electrification.

**Disclosure:**

No significant relationships.

